# Crystallization and Polymorphism of Organic Semiconductor in Thin Film Induced by Surface Segregated Monolayers

**DOI:** 10.1038/s41598-017-18881-y

**Published:** 2018-01-11

**Authors:** Seiichiro Izawa, Kyohei Nakano, Kaori Suzuki, Yujiao Chen, Tomoka Kikitsu, Daisuke Hashizume, Tomoyuki Koganezawa, Thuc-Quyen Nguyen, Keisuke Tajima

**Affiliations:** 1 RIKEN Center for Emergent Matter Science (CEMS), 2-1 Hirosawa, Wako, Saitama, 351-0198 Japan; 2 Center for Polymers and Organic Solids, University of California, Santa Barbara, CA 93106 USA; 3 Japan Synchrotron Radiation Research Institute (JASRI), 1-1-1 Kouto, Sayo-cho, Sayo, Hyogo, 679-5198 Japan; 4 Precursory Research for Embryonic Science and Technology (PRESTO), Japan Science and Technology Agency, 4-1-8 Honcho, Kawaguchi, Saitama, 332-0012 Japan; 5 Present Address: Institute for Molecular Science, 5-1 Higashiyama, Myodaiji, Okazaki, Aichi 444-8787 Japan

## Abstract

Preparation of highly crystalline organic semiconductor films is vital to achieving high performance in electronic devices. Here we report that surface segregated monolayers (SSMs) on top of phenyl-C_61_-butyric acid methyl ester (PCBM) thin films induce crystal growth in the bulk, resulting in a dramatic change in the structure to form a new crystal phase. Highly ordered crystalline films with large domain sizes of several hundreds of nanometers are formed with uniaxial orientation of the crystal structure perpendicular to the substrate. The molecular rearrangements in SSMs trigger the nucleation at a lower temperature than that for the spontaneous nucleation in PCBM. The vertical charge mobility in the SSM-induced crystal domains of PCBM is five times higher than in the ordinary polycrystalline domains. Using surface monolayers may be a new strategy for controlling crystal structures and obtaining high-quality organic thin films by post-deposition crystallization.

## Introduction

Highly ordered crystalline thin films are required for organic electronics devices such as organic field effect transistors (OFETs) and organic photovoltaics (OPVs) because the high order of the semiconducting molecules improves charge transport^1,2^ exciton diffusion^3,4^, and charge separation^5,6^. Separate control of the crystal nucleation and growth processes is difficult for thin films because the crystallization often occurs during evaporation of the solvents during the coating process. Complicated dynamic approaches have been reported to control these processes and obtain large crystalline domains. Minemawari *et al*. used injections of good and poor solvents for the semiconductors in inkjet printing to control the crystallization process^7^. In combination with pre-patterned substrates, the position of the nucleation site was controlled, resulting in the formation of single-crystalline films. Nakayama *et al*. reported the preparation of films for highly crystalline organic semiconductors by using a hot solution process^8^. The flow of the solution was controlled by the steady and slow movement of a slanted gap containing the solution, resulting in the oriented growth of single-crystalline films. Treat *et al*. reported small amount of a nucleating agent in the solution of organic semiconductors could control the solidification kinetics^9^. Heterogeneous nucleation increased the temperature and the rate of the crystallization, allowing the patterning of crystallites and minimizing dewetting of the films. The interactions between the molecules and the substrate surface also play an important role in controlling the crystal structures and their orientation in thin films, known as template (or epitaxial) growth. This method has been widely used in inorganic semiconductors and has been also investigated for organic semiconductors^10,11^. For example, orientations of phthalocyanines in vacuum-deposited films can be controlled by the choice of substrate such as graphene or CuI^12–14^. However, the choice of substrate is limited for device applications because the substrates also need to function as electrodes, dielectric layers, or charge transport layers, depending on the target electronic devices. Furthermore, template effects have been limited to films prepared by vacuum deposition^10^.

**Figure 1:**
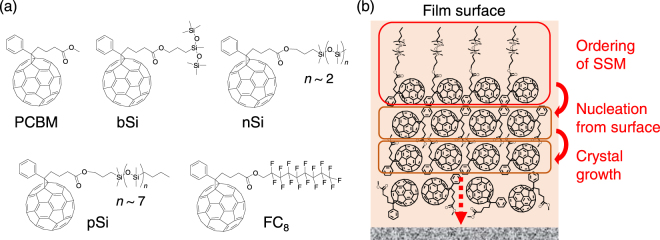
(**a**) Chemical structures of PCBM and the surface modifiers and (**b**) a schematic illustration of the crystallization induced by the pSi SSM.

We have been developing surface segregated monolayers (SSMs) as a facile method for modifying the surface of organic semiconducting films. SSMs can be prepared from a blend solution of semiconductors and surface modifiers. The surface modifier consists of the same or the similar organic semiconductor part that is covalently attached to a moiety with low surface energy such as fluoroalkyl chains^15–17^. Driven by minimizing the total energy, the surface modifiers spontaneously form a monolayer on the top surface of the semiconducting films (not on the substrate side) during the coating. SSMs have been used to modify the donor/acceptor interface of planar heterojunction OPVs^18–20^ or to achieve unique molecular orientations at the surface of the films^21–23^. In this study, we hypothesized that a self-organized SSM could act as a template layer for the crystallization of semiconducting materials in the bulk of the films (Fig. 1b). Similar to the organic film/substrate interface, the surface of the films could be another nucleation site for controlling the crystallization in the bulk of the films, but free from the limitation in the choice of substrates. Note that this surface-induced crystallization can be significantly different from the conventional substrate-induced crystallization in term of the nucleation mechanism, since the molecules are much more mobile at the surface than at the substrate interfaces. The role of SSMs in the crystal growth of an organic semiconductor in thin films and their effects on the electronic conduction properties were investigated.

## Results and Discussion

### Synthesis of new surface modifiers, evidence of SSM

Previously, we reported that well-ordered SSMs of a fullerene derivative with semifluoroalkyl chains (FC_8_ in Fig. 1a) formed on the surface of [6,6]-phenyl-C_61_-butyric acid methyl ester (PCBM) films^16^. PCBM is an *n*-type organic semiconductor extensively studied for OPVs^24^ and OFETs^25^. In this work, we synthesized new fullerene derivatives with various oligosiloxane chains as surface modifiers and crystallization inducers (pSi, bSi, and nSi in Fig. 1a). Oligosiloxanes have a low surface energy similar to fluoroalkyl chains but a weaker tendency to crystallize owing to the flexibility of the chains^26^. Therefore, the derivatives could form SSMs on PCBM films with the flexibility to adopt a suitable molecular orientation for the crystallization of PCBM. The synthesis and characterization of the derivatives are described in the Supplementary Information.

**Figure 2:**
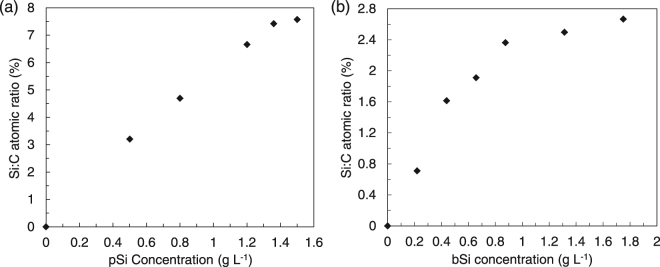
Si/C atomic ratio on the surfaces of the films in (**a**) pSi/PCBM and (**b**) bSi/PCBM films measured by XPS plotted as a function of the modifier concentrations in the solution. All the films were spin-coated on silicon wafers. The solution contains a fixed concentration of PCBM (10 g L^−1^).

The surface segregation behaviors of the molecules in the films were investigated by X-ray photoelectron spectroscopy (XPS) according to the previously established method for the fluoroalkylated molecules^15,17,19^. The films were prepared by spin-coating blend solutions of the surface modifier and PCBM with different concentrations of pSi (0.50–1.50 g L^−1^), bSi (0.22–1.76 g L^−1^), and nSi (0.22–1.76 g L^−1^), and a fixed concentration of PCBM (10 g L^−1^). The pSi/PCBM films were annealed under N_2_ at 130 °C for 30 min, and the bSi/PCBM and nSi/PCBM films were annealed at 160 °C for 10 min before XPS measurements. The Si/C atomic ratio at the surface was calculated from the intensities of the Si 2p and C 1 s peaks. Figure 2 shows the Si/C atomic ratio on the surface of the pSi/PCBM and bSi/PCBM films as a function of the concentration of the surface modifiers in the solutions. The results for nSi/PCBM are presented in Figure S3. The dependence of the surface atomic components on the concentrations are similar to those of our previous studies on the SSM formation in FC_8_/PCBM. Clear saturation of the Si/C ratios was observed at high surface modifier concentrations in all cases, indicating that they segregated to the surface of PCBM films^16^. The concentration of the surface modifiers close to the saturation point (1.36 g L^−1^ for pSi, 0.88 g L^−1^ for bSi, and 1.32 g L^−1^ for nSi) were used for investigating the film properties further.

We also performed static water contact angle measurements, XPS depth profiles, angle-resolved XPS, and elemental mapping by scanning transmission electron microscopy (STEM) of the films. The results indicated that the oligosiloxane chains of pSi, bSi, and nSi were uniformly dispersed at the surface of the PCBM films. The surface coverage with the surface modifier at the selected saturation points was estimated as higher than 90% in the bSi/PCBM film based on the lower-limit calculation assuming a simple bilayer model^27^. The details of the surface analysis are provided in the Supplementary Information.

### Differences in crystal structure

**Figure 3:**
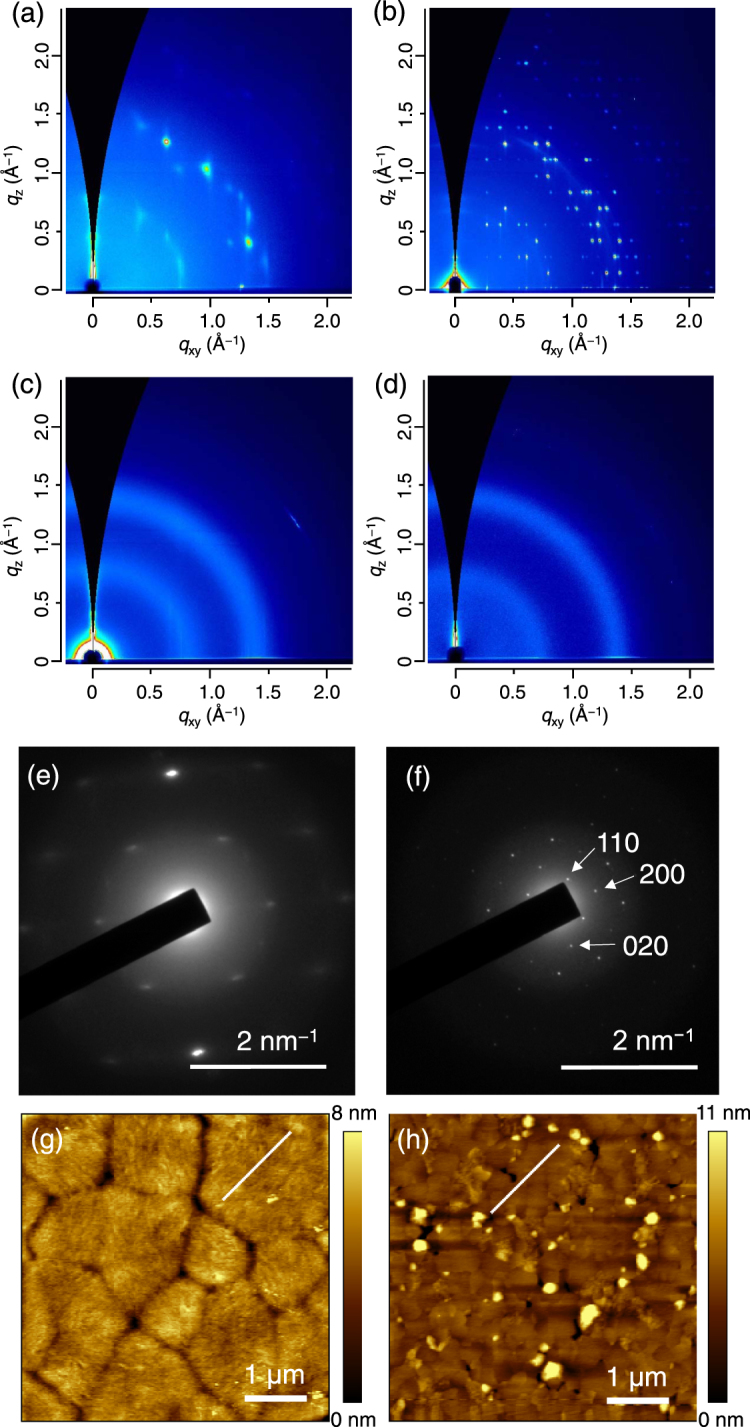
GIWAXS patterns of (**a**) PCBM, (**b**) pSi/PCBM, (**c**) pSi and (**d**) non-annealed PCBM films on silicon wafers. SAED pattern of (**e**) PCBM and (**f**) pSi/PCBM films taken by TEM. The diameter of the selected area is 230 nm. AFM height images of (**g**) pristine PCBM and (**h**) pSi/PCBM films. Line profiles along the white lines are shown in Figure S11. The pSi/PCBM film were prepared by spin-coating the mixed solution of pSi (1.36 g L^−1^) and PCBM (10 g L^−1^). PCBM and pSi films were thermally annealed at 160 °C for 10 min and pSi/PCBM film was annealed at 130 °C for 30 min.

Figure 3a and b show two-dimensional grazing incidence wide-angle X-ray scattering (GIWAXS) patterns of the pristine PCBM films and pSi/PCBM films (pSi concentration: 1.36 g L^−1^) after thermal annealing. The PCBM film showed Bragg spots corresponding to the polycrystalline thin-film phase, which have been observed after cold crystallization^28,29^. In contrast, the pSi/PCBM film pattern showed a number of sharp Bragg spots that were completely different from the annealed pristine PCBM film pattern. The critical angle of the PCBM film calculated by its density is 0.115°, therefore GIWAXS patterns measured at an incident angle of 0.12° reflects the diffraction from the crystal in the bulk of the thin films (details in the Supplementary Information). The patterns with the smaller incident angles showed smaller peak intensities without any change of the pattern, indicating that the crystal structure is not only in the surface region but in the bulk of the film (Figure S7). The GIWAXS pattern of the pSi/PCBM film did not match either the reported single-crystal phases or thin film phase of PCBM^29–32^. Because the pristine pSi film is amorphous after annealing at 160 °C showing only halos in the GIWAXS pattern (Fig. 3c), the sharp diffraction spots in pSi/PCBM were attributed to a new phase of the PCBM crystal in the bulk of the film. In addition, the pSi/PCBM film showed almost no halos that come from the amorphous PCBM at *q* = 0.69 and 1.36 Å^−1^ (see Fig. 3d for the GIWAXS pattern of the as-cast PCBM film), indicating that the crystallinity of the films was much higher than that of the pristine PCBM film after annealing. GIWAXS patterns with the different surface modifiers (bSi, nSi, and FC_8_) showed the same pattern as in pSi/PCBM (Figure S8a–c); however, the crystallization temperature, diffraction intensity of the films, and the phase purity were different as we discuss below.

Selected area electron diffraction (SAED) measurements are shown in Fig. 3e and f. The pSi/PCBM film showed sharp diffraction spots, whereas the pristine PCBM films showed broader spots. There was no directional variation in the diffraction patterns of the pSi/PCBM films, suggesting that the size of the single-crystalline domains was larger than the aperture size (~230 nm) of the SAED measurements. Transmission electron microscopy (TEM) images of the pSi/PCBM films are shown in Figure S9. Because the pSi/PCBM films were highly crystalline, a crystal lattice was observed, whereas no pattern was visible in the pristine PCBM film after annealing. The crystal size in the pSi/PCBM films was in the range of several hundreds of nanometers. The mean size of the crystalline domains in the pSi/PCBM film was estimated as larger than 185 nm from the GIWAXS pattern by using the Scherrer equation (Supplementary Information). This results is in the same order with the observed crystal domain size in TEM image (Figure S9).

The surface morphology of the crystallized films was investigated by AFM (Fig. 3g and h). The pristine PCBM film showed a surface with the root mean square (RMS) of 0.94 nm for the whole image and grains 1–2 μm in size separated by clear grain boundaries visible as grooves. Each grain had a fine surface texture and the RMS of the line profile in Fig. 3g was 0.47 nm (see Figure S11). In contrast, pSi/PCBM films had a surface morphology with texture-less domains and some protrusions and holes in the films several hundred nanometers in size, which may have formed during the crystallization. The polygonal shape of the grains was visible, but the boundaries were less clear than in the pristine PCBM films. RMS for the whole image was 1.3 nm, although the grain surfaces were very flat and the RMS of the line profile in Fig. 3h was 0.26 nm (Figure S11). The differences in the film morphology imply that the presence of the SSM induced a large change in the nucleation and growth processes of the crystals.

### Crystal structure analysis

The pSi/PCBM films showed many well-aligned Bragg spots in the GIWAXS patterns, indicating the formation of highly ordered single-crystal domains in the SSM-induced phase. The crystal system and the cell constants of the phase were determined certainly as a body-centered tetragonal unit-cell with *a* = *b* = 32.73, *c* = 45.55 Å, *V* = 48795.6 Å^3^ from the GIWAXS, out-of-plane XRD, and SAED patterns^33^. The *c*-axis was oriented perpendicular to the surface of the films, whereas the *a*- and *b*-axes are oriented in the plane. The mosaicity of the crystal in the out-of-plane direction was estimated to be 0.95°−1.62°, indicating that the orientation order of the crystal is very high in the vertical direction of the films (see Supplementary Information). The cell volume of the SSM-induced phase is about 12 times larger than that of the solvent-free PCBM single-crystal with a monoclinic unit cell and *Z* = 4^30,31^, therefore *Z* = 48 can be assumed. The calculated density of the SSM-induced crystal based on this assumption is 1.49 g cm^−3^, similar to the bulk density of PCBM (~1.5 g cm^−3^) but smaller than the reported values for the single crystals (1.631–1.653 g cm^−3^).

**Figure 4:**
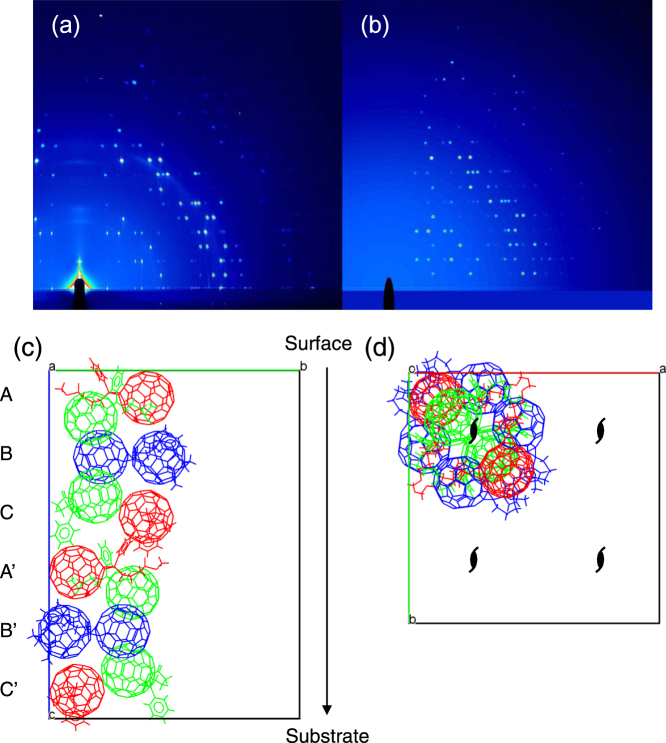
(**a**) As-recorded GIWAXS pattern of the annealed pSi/PCBM film and (**b**) simulated GIWAXS pattern from the solved crystal structure for the same measurement conditions. Views of the packing of PCBM along (**c**) the *a*-axis and (**d**) the *c*-axis with only one of the four columns along the 2-fold screw axes in the unit cell displayed. The molecules are colored according to their symmetrical equivalence.

To gain insights into the crystallization process in the film, we analyzed the diffraction data to determine the packing structure of the SSM-induced films (see Supplementary Information for the details). The Bragg spots in the GIWAXS patterns and the out-of-plane XRD data were indexed and integrated to produce *hkl*-*I* datasets. The positions and the conformations of the three independent PCBM molecules in the unit cell were explored by a direct-space method with a simulated annealing algorithm implemented in SIR2014 software^34^. We obtained a solution having *I* $\bar{4}$ *c*2 space group with three crystallographically independent PCBM molecules with the lowest figure of merit. The structure was refined by a least-squares method with SHELXL-2016/6 software (CIF attached)^35^. The simulated GIWAXS pattern from the optimized structure reproduces the observed pattern well (Fig. 4a and b). The crystal structure is constructed with the repetition of six layers (A, B, C, A’, B’ and C’) along the *c*-axis in the lateral view (Fig. 4c) and four columns along the four 2-fold screw axes in the top view (Fig. 4d). Each layer is formed through the interaction between closely packed fullerene moieties in the *ab*-plane. A, C, A’ and C’ layers consist of two of three crystallographically independent molecules (red and green). Neighboring independent molecules contact with each other in a zigzag manner. B and B’ layers consist with the other independent molecules (blue) that pack almost planar in the *ab*-plane. The layers stack in the order of A-B-C-A’-B’-C’. The orientations of the phenyl substituents in each layer are either upward (A and A’), downward (C and C’) or both (B and B’). This switching of the substituent directions may be originating from the preferable fullerene-fullerene interaction. On the other hand, the interactions between the phenyl butyric acid methyl esters may specifically determine the relative molecular orientations in the next layer, resulting in the exceptionally long repeating unit in the direction of the *c*-axis. These results suggest that the SSM with the preferred molecular orientation at the surface (i.e. the siloxane ester substitution pointing to the surface) could trigger the packing of PCBM beneath through the fullerene-fullerene interactions and the growth of the crystal in the bulk of the films. The unusually large unit-cell may be related to the unique planar nucleation from the oriented fullerene layer leading to the metastable local packing being transferred from the surface into the bulk.

### Evidence of induced crystallization by SSM

**Figure 5:**
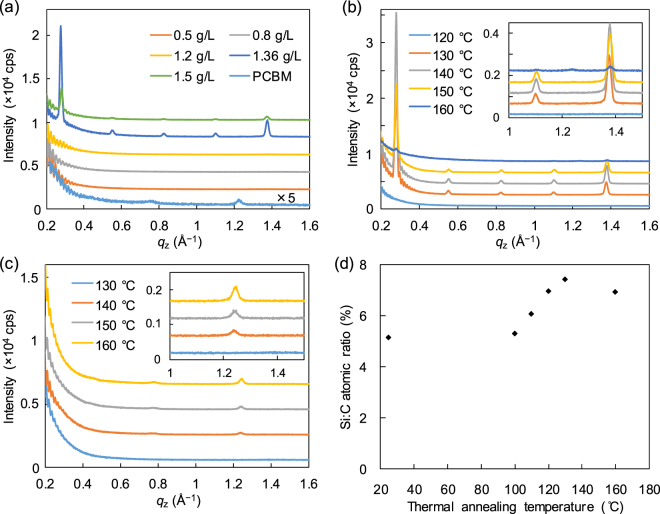
(**a**) Out-of-plane XRD pattens of the pristine PCBM film and the pSi/PCBM films with different surface modifier concentrations. All the films were spin-coated on silicon wafers. The pristine PCBM film was thermally annealed at 150 °C and the pSi/PCBM films were annealed at 130 °C. The patterns are shifted along the *y*-axis for clarity. Out-of-plane XRD patterns of (**b**) pSi/PCBM and (**c**) pure PCBM films on silicon wafers, which were thermally annealed at different temperatures. The bSi/PCBM film were prepared by spin-coating the mixed solution of bSi (0.88 g L^−1^) and PCBM (10 g L^−1^). The insets show magnified views. The patterns are shifted in the *y*-axis for clarity. (**d**) Si:C atomic ratio on the surfaces of the pSi/PCBM films measured by XPS plotted as a function of the thermal annealing temperature. The films with SSM were prepared by spin-coating the mixed solution of PCBM (10 g L^−1^) and the surface modifiers. The concentration of pSi was 1.36 g L^−1^.

Figure 5a shows out-of-plane XRD patterns of the pSi/PCBM films after annealing at 130 °C for 30 min with different concentrations of the surface modifier in the solution. There was a threshold concentration of pSi in the spin-coating solution for the formation of the new crystal phase; when the concentration of pSi in solution was larger than 1.36 g L^−1^, clear diffraction peaks at 0.28, 0.56, 0.85, 1.11, and 1.39 Å^−1^ appeared (*d*-spacing: 22.6, 11.3, 7.55, 5.67, and 4.54 Å, respectively, and the index: 002, 004, 006, 008, and 0010, respectively). The peak positions were different from those in the pristine PCBM films after annealing (the small peaks at 0.79 and 1.25 Å^−1^, *d*-spacing: 8.26 and 5.09 Å, respectively, see also the inset). This threshold concentration agreed well with the saturation concentration for Si/C ratios measured by XPS (Fig. 2a), suggesting that the SSM begins to induce the crystallization of PCBM when the film surface is sufficiently covered with the pSi monolayer. Using indium tin oxide (ITO)-coated glass or glass as the substrate instead of silicon wafer did not change the diffraction patterns, suggesting that the induced crystallization is insensitive to the substrates (Figure S8d and e). From these results, we concluded that the new crystal phase of PCBM was induced by the presence of SSMs.

Next, we examined the effects of the annealing temperature on the crystallization behaviors. Figure 5b shows out-of-plane XRD patterns of pSi/PCBM film after thermal annealing at various temperatures. The peaks at 0.28, 0.56, 0.85, 1.11, and 1.39 Å^−1^ appeared after annealing above 130 °C, indicating the formation of the pure SSM-induced phase. When the films were annealed above 160 °C, the small peaks of the normal crystal phase of PCBM also appeared at 0.79 and 1.25 Å^−1^. The intensity of the peaks for the SSM-induced phase decreased at 150 °C, and the normal phase was observed as the main phase at 160 °C. The growth of the SSM-induced phase started at a lower temperature (130 °C) than the spontaneous nucleation of PCBM (140 °C) (Fig. 5c) and previously reported hexagonal ordering (280 °C)^32^. These results indicated that the SSM facilitates the crystallization of PCBM into the new phase.

The crystallization behaviour of nSi/PCBM films is similar to that of pSi/PCBM with a crystallization temperature of 130 °C (Figure S12). However, bSi/PCBM and FC_8_/PCBM films showed a higher crystallization threshold temperature of 140 °C. These results may suggest that the transition temperature depends on the chemical structure and mobility of the surface modifiers at the surface. To confirm this hypothesis, the surface Si/C ratios of pSi/PCBM films after thermal annealing at different temperatures were measured by XPS (Fig. 5d). The Si/C ratio gradually increased by 40% as the annealing temperature increased from 100 to 130 °C, and was saturated above 130 °C, which is close to the transition temperature from amorphous PCBM to the SSM-induced crystal. The increase of the Si/C ratio was attributed to the change in the molecular orientation of pSi by exposing more oligosiloxane chains to the air. This suggests that the crystallization of PCBM in pSi/PCBM films happens after the rearrangement of the surface structure. In contrast, in FC_8_/PCBM films that showed a higher crystallization temperature of 140 °C and much smaller diffraction peaks compared with the oligosiloxane-based SSM molecules, the increase of F/C ratios at the surface caused by thermal annealing was smaller (9% at 130 °C) and the saturation happened at a higher temperature (Figure S13). This was attributed to strong interactions between the fluoroalkyl chains, which can prevent the rearrangement of the molecules at the surface. These results suggest that the flexibility of the oligosiloxane chains and the surface reorientation of the molecules are important for induced crystallization from SSM.

**Figure 6:**
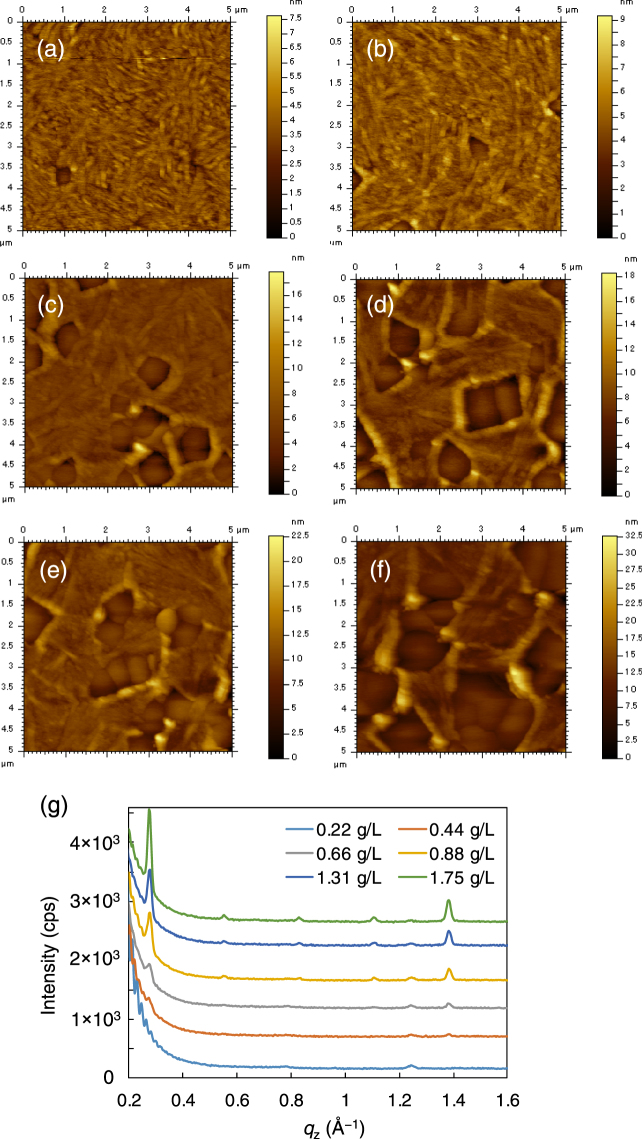
AFM height images of the bSi/PCBM films on silicon wafers with bSi concentrations of (**a**) 0.22 g/L, (**b**) 0.44 g/L, (**c**) 0.88 g/L, (**d**) 0.66 g/L, (**e**) 1.31 g/L, and (**f**) 1.75 g/L and a fixed PCBM concentration of 10 g L^−1^. (**g**) Out-of-plane XRD pattens of the bSi/PCBM films on silicon wafers with different surface modifier concentrations. The patterns are shifted in the y-axis for clarity. The films were spin-coated on silicon wafers and thermally annealed at 160 °C.

When the films were annealed at temperatures higher than 140 °C, at which spontaneous nucleation and PCBM crystallization start, the peaks from both the SSM-induced phase and the normal phase were visible in the XRD patterns. The coexistence of the two crystal phases was also observed in the AFM image in Fig. 6a–f. Two types of region with different roughnesses were observed: rough regions with a fine texture similar to that of the pristine PCBM film after crystallization; and textureless smooth domains with polygonal shapes. There were also interdomain regions that protruded from the other domains. The intensities of the diffraction peaks from the SSM-induced crystal in the XRD patterns and the area of the smooth surface simultaneously increased as the concentration of the surface modifier was increased (Fig. 6g). Based on these results, the domains with the textured surface in the films were assigned as the normal crystal phase of PCBM and the smooth polygonal domains were assigned as the SSM-induced crystals.

### Proposed crystallization mechanism

According to our observations, we propose a mechanism for the induced crystallization from SSM (Fig. 1b). During the thermal annealing, the SSM molecules change their orientation to expose more oligosiloxane chains to the air and lower the total energy of the system. This exposes the fullerene groups of the SSM molecules to the interface with PCBM. Subsequently, the crystallization of PCBM is nucleated from the interface with SSM and the growth proceeds vertically, resulting in the formation of large crystal domains and uniaxial orientation. The nucleation temperature at SSMs is lower than the spontaneous nucleation in the pristine PCBM films, resulting in the formation of the pure SSM-induced phase in pSi/PCBM and nSi/PCBM at the correct annealing temperature.

### Electrical conduction properties

**Figure 7:**
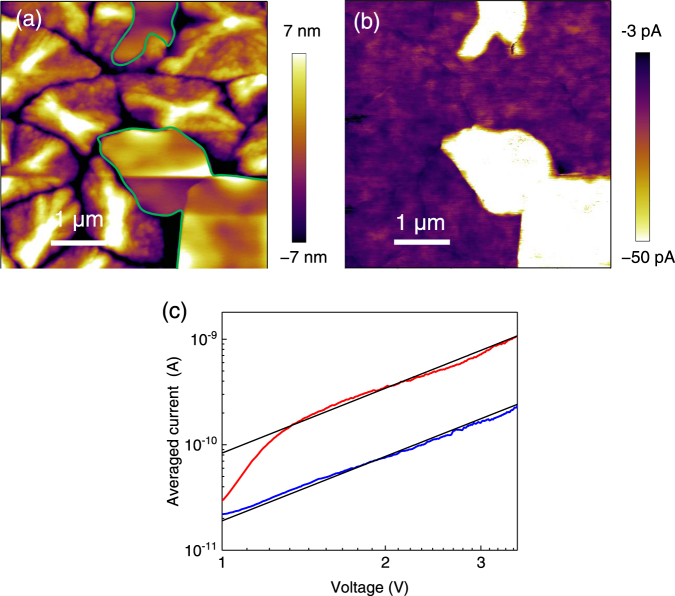
(**a**) Height image and (**b**) current image of the bSi/PCBM film measured by c-AFM after thermal annealing at 160 °C. The bSi/PCBM film were prepared by spin-coating the mixed solution of bSi (0.88 g L^−1^) and PCBM (10 g L^−1^). A bias of −1.5 V was applied to the ITO substrate. Areas with high flatness assigned as the SSM-induced phase are enclosed by the green lines. (**c**) Log *I* vs. log *V* plot in the normal crystal domain (blue line) and the SSM-induced crystal domain (red line) measured by c-AFM. Current was averaged at 10 different points in the domains, as shown in Figure S14. Black lines were fitted curve with a slope of 2 by using the modified SCLC model for c-AFM.

The electrical conduction properties of the normal and SSM crystals were investigated by conductive AFM (c-AFM) measurements. c-AFM can be used to obtain topography and current images simultaneously and to evaluate the electronic properties of the films, such as the nanoscale conductivity and charge mobility, without suffering from the effects of the lateral grain boundaries. Current images of the PCBM films were measured on ITO/polyethylenimine ethoxylated (PEIE)/PCBM films using an Au tip, and electrons flowed from the substrate to the tip when a negative bias was applied to the substrate^36^. The bSi/PCBM film sample was annealed at 160 °C, allowing the formation of both the SSM-induced phase and the normal phase of PCBM within the same sample. Thus, we could compare the differences in the electrical properties between the domains in the same film. Figure 7a and b show topographic and current images collected at −1.5 V, respectively, in the same field of view. In the topographic image, the SSM-induced domains were the flat regions (enclosed by green lines). In the current image, the current transport through the SSM-induced crystals domains was 5–7 times larger than that of the normal crystal domains, indicating that the conductivity of the SSM-induced crystal phase was much higher. Quantitative analysis of the electron mobility of two crystals was conducted by fitting *I*-*V* curves obtained by c-AFM to the space charge limited current (SCLC) model^37^. Fig. 7c shows a log *I* vs. log *V* plot measured in the two domains averaged at 10 different points and the fitting curves obtained by using the modified SCLC model. All the *I*-*V* curves and measuring spots are described in Figure S14. The slopes of the *I-V* curves were close to 2, which is characteristic of the SCLC region (Table S2). The intercept of the fitting curve of the SSM-induced crystal was 5 times larger than that of the normal crystals, indicating that the electron mobility of SSM crystal was 5 times higher because the thicknesses were the same. The average mobility in the domains was evaluated by using the modified SCLC model for c-AFM for electron injection from the substrate. The tip-sample contact area was estimated by using the Hertz model^38^. The details of the model and estimation are described in the Supplementary Information. The electron mobility values in the SSM-induced crystal and the normal crystal domains were 0.050 and 0.011 cm^2^ V^−1^ s^−1^, respectively, whereas the reported electron mobility values in PCBM films measured by electron-only diode SCLC are 2 × 10^−3^–5 × 10^−3^ cm^2^ V^−1^ s^−1^ ^39–41^. The mobility values measured by c-AFM are frequently overestimated compared to the bulk mobility due to the difficulty of estimating the tip-sample contact area^42^; however, it was clear that the electron mobility in the SSM-induced crystal phase was much larger than that in the normal crystal phase of PCBM.

## Conclusions

In summary, we demonstrated that the nucleation from SSMs in PCBM films lowered the transition temperature to the crystal phase and also induced marked polymorphism. The highly ordered crystalline films showed higher electronic conduction properties in the vertical direction. The concept of SSM-induced crystallization in thin films can be generalized to other materials such as donor molecules or semiconducting polymers. It is a promising strategy for controlling the crystal structure in thin films for organic electronic devices because it is a solvent-free, substrate-independent, post-deposition crystallization that should be compatible with many solution processes.

## Methods

### Sample preparation

Si wafers, ITO-coated glass substrates and glass substrates were cleaned by sequential ultrasonication in detergent solution, water, acetone, and 2-propanol. The substrates were dried, and then exposed to UV-O_3_ for 30 min. The spin-coating solution was prepared by dissolving PCBM (10 mg) and surface modifiers (bSi: 0.22–1.76 mg; nSi: 0.22–1.76 mg; pSi: 0.5–1.5 mg; FC_8_: 1 mg) in chloroform (1 mL). The solution was spin-coated at 2500 rpm for 30 s. The film thickness was about 40 nm. The films were thermally annealed under N_2_. Samples for the c-AFM measurement were prepared on a PEIE/ITO substrate. The PEIE solution for spin-coating was prepared by mixing 80% PEIE solution (10 µL) and 2-methoxyethanol (2.49 mL) at room temperature. The PEIE solution was spin-coated at 5000 rpm for 60 s on a pre-cleaned ITO substrate. The PEIE/ITO film was thermally annealed at 100 °C for 10 min under N_2_.

### Materials and instruments

All chemicals were purchased from chemical suppliers (Sigma-Aldrich, Tokyo Kasei Kogyo, Wako Chemicals, or Gelest) and used without further purification. ^1^H NMR spectra were measured with an NMR spectrometer (JNM-AL300, JEOL). MALDI-TOF-MS was performed with a mass spectrometer (Ultraflex RO, Bruker Daltonics) in negative ion mode with dithranol as the matrix. X-ray photoelectron spectroscopy (XPS) was performed with a surface analysis instrument (PHI 5000 Versa Probe II, ULVAC-PHI). Monochromated Al Kα (1486.6 eV) radiation was used in all XPS measurements. The C 1 s (282 eV), and Si 2p (102 eV) peaks were used for the characterizations. To obtain the XPS depth profile, each sample was etched with Ar^+^ at an acceleration voltage of 500 V with an etching rate of approximately 0.25 nm s^−1^. Film thickness was measured by using a surface profilometer (Dektak 6 M, ULVAC). The static contact angle of a droplet of distilled water on the each film was measured with a contact angle meter (DMe-201, Kyowa) at room temperature. Two-dimensional grazing incidence wide-angle X-ray scattering (GIWAXS) patterns were measured at an incident angle of 0.12° by using the synchrotron radiation at beamline BL46XU of SPring-8 with the approval of the Japan Synchrotron Radiation Research Institute (JASRI). Out-of-plane X-ray diffraction (XRD) patterns with *θ*/2*θ* scan were measured with an X-ray diffractometer (Smart Lab, Rigaku) with Cu Kα at 45 kV and 200 mA. Transmission electron microscopy (TEM), selected area electron diffraction (SAED), and elemental mapping by energy dispersive X-ray spectrometry were performed with a transmission electron microscope (JEM-2100F, JEOL) with an acceleration voltage of 200 kV. The diameter of the aperture for the selected area was 230 nm. The PCBM film sample was transferred onto a Cu grid with a carbon supporting film by using CYTOP (AGC, Japan) as a support layer. Atomic force microscopy (AFM) images were obtained with a scanning probe microscope (5400, Agilent Technologies) in tapping mode. Conductive AFM (c-AFM) current images and the nano *I–V* curves were collected in the dark using an atomic force microscope (MFP-3D, Asylum Research) under an N_2_ atmosphere. Gold-coated silicon AFM tips (Budget Sensors) were used with a resonant frequency of 13 kHz and a force constant of 0.2 N m^−2^.

### Data Availability

All data generated or analyzed during this study are included in this published article (and its Supplementary Information files).

## Electronic supplementary material

Supplementary Information

Crystal structure for PCBM film
