# Supplementary material for: Crystallization and Polymorphism of Organic Semiconductor in Thin Film Induced by Surface Segregated Monolayers
Source: Sci Rep. 2018 Jan 11;8:481. doi: 10.1038/s41598-017-18881-y (PMC5764981; doi:10.1038/s41598-017-18881-y)
Supplement: Supplementary file 1 — Supplementary Information [file 41598_2017_18881_MOESM1_ESM.pdf]

Supplementary Information for

Crystallization and Polymorphism of Organic Semiconductor in Thin Film Induced by Surface Segregated Monolayers

*Seiichiro Izawa[1,2†], Kyohei Nakano[1], Kaori Suzuki[1],Yujiao Chen[1], Tomoka Kikitsu[1], Daisuke Hashizume[1], Tomoyuki Koganezawa[3], Thuc-Quyen Nguyen[2] and Keisuke Tajima[1,4]**

[1]RIKEN Center for Emergent Matter Science (CEMS), 2-1 Hirosawa, Wako, Saitama 351-0198, Japan

[2]Center for Polymers and Organic Solids, University of California, Santa Barbara, CA 93106, USA

[3]Japan Synchrotron Radiation Research Institute (JASRI), 1-1-1 Kouto, Sayo-cho, Sayo, Hyogo 679-5198, Japan

[4]Precursory Research for Embryonic Science and Technology (PRESTO), Japan Science and Technology Agency, 4-1-8 Honcho, Kawaguchi, Saitama 332-0012, Japan

†Current address: Institute for Molecular Science, 5-1 Higashiyama, Myodaiji, Okazaki, Aichi, 444-8787, Japan

E-mail: keisuke.tajima@riken.jp

Synthesis of oligosiloxane fullerene derivatives

The synthetic route to the surface modifiers with oligosiloxane is shown in Scheme S1. The details of the synthetic procedures, and the spectral data from ^{1}H NMR spectroscopy and MALDI-TOF-MS, are described below.

General synthesis of 3-hydroxy-propyl-terminated oligodimethylsiloxane

1,1,1,3,5,5,5-Heptamethyltrisiloxane, 1,1,1,3,3,5,5-heptamethyltrisiloxane, or n-butyl/hydride-terminated polydimethylsiloxane (Mw: 800–900) (22.5 mmol), allyl alcohol (1.54 mL, 22.5 mmol), platinum(0)-1,3-divinyl-1,1,3,3-tetramethyldisiloxane complex solution (200 μL), and toluene (10 mL) were added to a 50 mL round-bottom flask. The reaction mixture was stirred at room temperature for 1 day. The reaction mixture was purified by silica gel column chromatography using chloroform as the eluent. The solvent was removed under vacuum, yielding the product as a colourless transparent liquid.

3-(3-Hydroxy-propyl)-1,1,1,3,5,5,5-heptamethyltrisiloxane (yield, 47%)

^{1}H NMR ($CDCl_3$, 300 MHz): δ (ppm): 3.52–3.60 (m, 2H), 1.52–1.63 (m, 2H), 0.58–0.66 (m, 2H), 0.06–0.12 (m, 21H).

5-(3-Hydroxy-propyl)-1,1,1,3,3,5,5-heptamethyltrisiloxane (yield, 70%)

^{1}H NMR ($CDCl_3$, 300 MHz): δ (ppm): 3.59–3.63 (t, 2H), 1.56–1.66 (m, 2H), 0.52–0.58 (m, 2H), 0.02–0.11 (m, 21H).

3-Hydroxy-propyl, n-butyl-terminated polydimethylsiloxane (yield, 34%)

^{1}H NMR ($CDCl_3$, 300 MHz): δ (ppm): 3.56–3.62 (m, 2H), 1.56–1.66 (m, 2H), 1.27–1.33 (m, 4H),

0.86–0.90 (t, 3H), 0.51–0.57 (m, 4H), 0.03–0.10 (m, 69H).

Synthesis of bSi, nSi, and pSi

3-[1,3,3,3-Tetramethyl-1-[(trimethylsilyl)oxy]-1-disiloxanyl]-1-propanol, 3-(1,1,3,3,5,5,5-heptamethyl-1-trisiloxanyl)-1-propanol, or n-butyl/hydroxypropyl-terminated polydimethylsiloxane (5.49 mmol), [6,6]-phenyl-C_{61}-butyric acid methyl ester (PCBM) (500 mg, 0.549 mmol), *p*-toluenesulfonic acid monohydrate (115 mg, 6.04 mmol), and anhydrous *o*-dichlorobenzene (25 mL) were added to a 50 mL two-neck round-bottom flask under N_2. The reaction mixture was stirred at 180 °C for 1 day and then poured into methanol (200 mL). The resulting solid was collected by vacuum filtration and the crude product was purified by silica gel column chromatography using toluene as the eluent. The product was reprecipitated in methanol and filtered, yielding a brown solid.

bSi (yield, 12%)

^{1}H NMR ($CDCl_3$, 400 MHz): δ (ppm): 7.92–7.94 (m, 2H), 7.53–7.56 (m, 2H), 7.47–7.49 (m, 1H), 4.00–4.04 (t, 2H), 2.90–2.94 (m, 2H), 2.50–2.54 (m, 2H), 2.18–2.21 (m, 2H), 1.59–1.67 (m, 2H), 0.44–0.48 (m, 2H), 0.02–0.12 (m, 21H). MALDI-TOF-MS: calcd. 1159.4, found 1157.8.

nSi (yield, 40%)

^{1}H NMR ($CDCl_3$, 300 MHz): δ (ppm): 7.92–7.94 (m, 2H), 7.52–7.57 (m, 2H), 7.44–7.49 (m, 1H), 4.01–4.05 (t, 2H), 2.89–2.94 (m, 2H), 2.49–2.54 (m, 2H), 2.14–2.24 (m, 2H), 1.58–1.70 (m, 2H), 0.48–0.56 (m, 2H), 0.01–0.09 (m, 21H). MALDI-TOF-MS: found 1083.9, 1157.9, 1232.9, 1309.1 (mixture of oligomers).

pSi (yield, 34%)

^{1}H NMR ($CDCl_3$, 300 MHz): δ (ppm): 7.92–7.94 (m, 2H), 7.52–7.57 (m, 2H), 7.43–7.49 (m, 1H), 4.00–4.05 (t, 2H), 2.89–2.94 (m, 2H), 2.49–2.54 (m, 2H), 2.16–2.21 (m, 2H), 1.56–1.69 (m, 2H), 1.29–1.33 (m, 4H), 0.86–0.90 (m, 3H), 0.50–0.56 (m, 4H), 0.02–0.09 (m, 53H). MALDI-TOF-MS: found 1126.0, 1201.0, 1275.0, 1423.0, 1497.0 (mixture of oligomers).

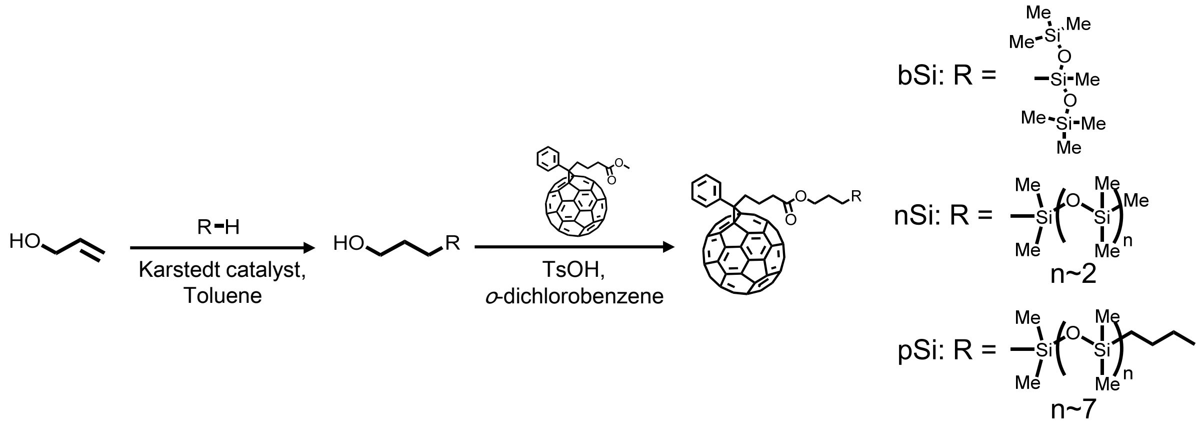

Scheme S1. Synthesis route of the surface modifiers (bSi, nSi, and pSi).

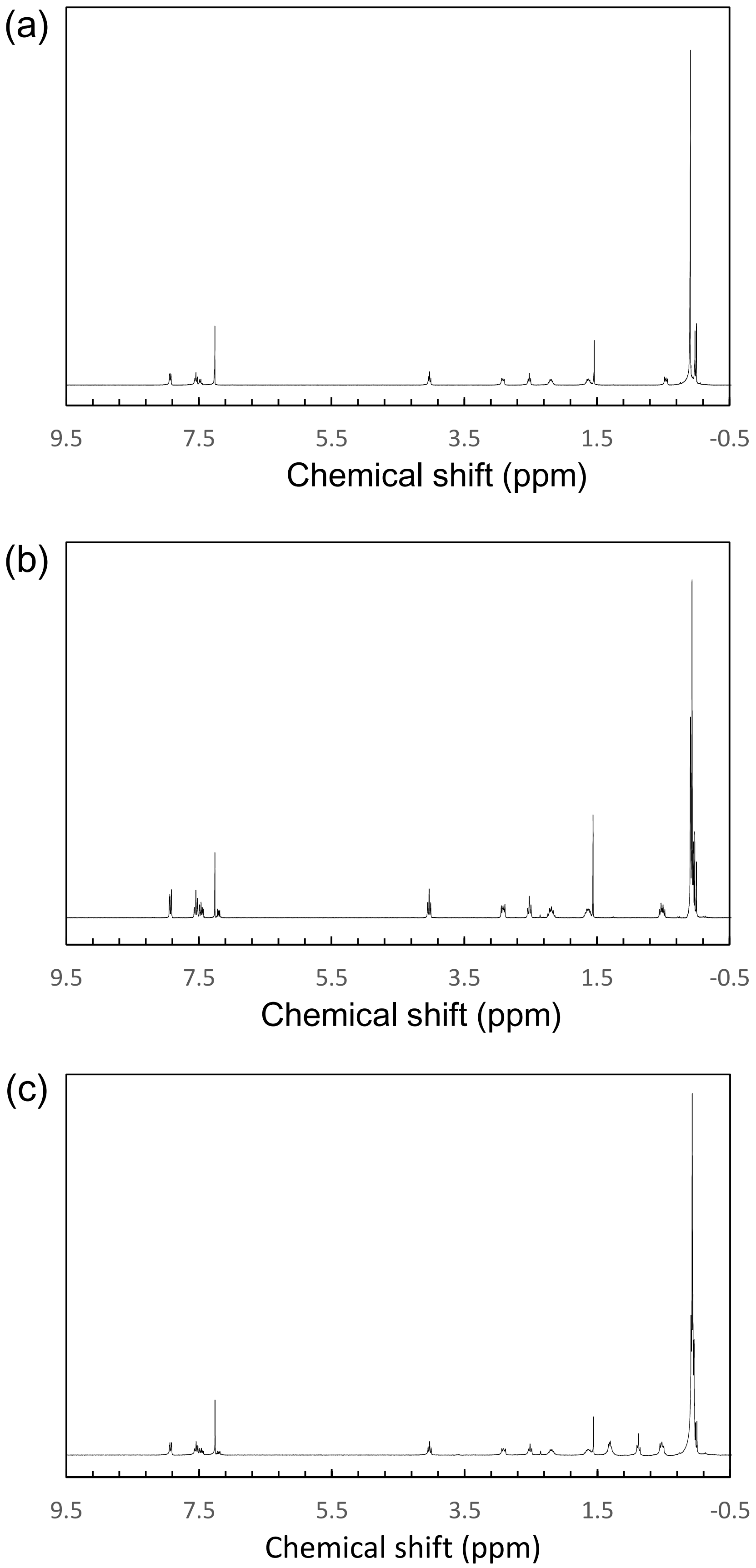

Figure S1. ^{1}H NMR spectra of (a) bSi, (b) nSi, and (c) pSi in $CDCl_3$.

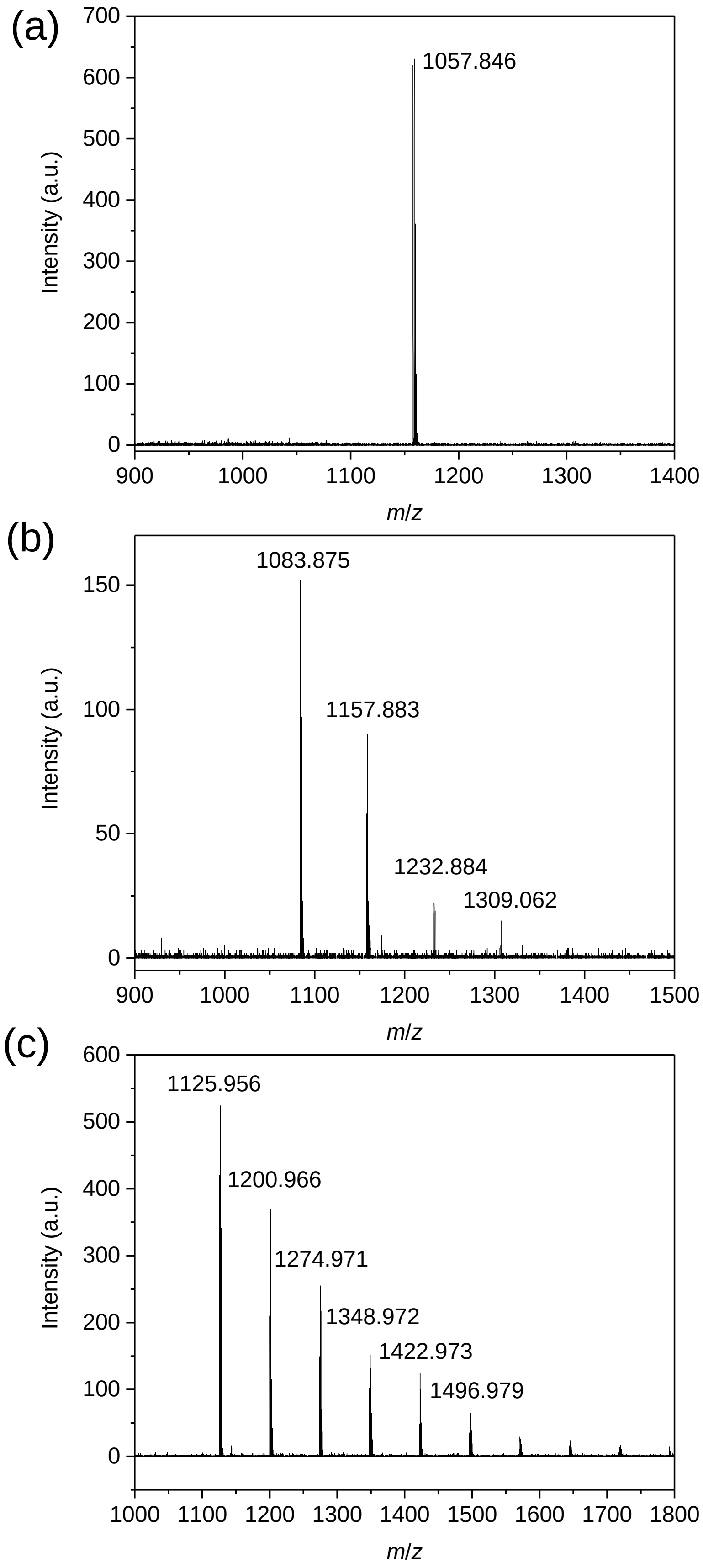

Figure S2. MALDI-TOF-MS charts of (a) bSi, (b) nSi, and (c) pSi.

Si:C atomic ratios on the film surfaces measured by XPS

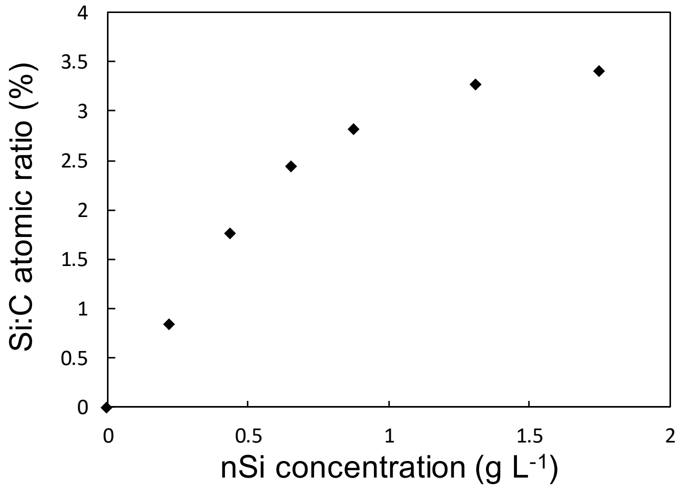

Figure S3. Si/C atomic ratios on the film surfaces measured by XPS plotted as a function of the nSi concentrations in the spin-coating solution. The solutions contain a fixed concentration of PCBM (10 g L^{-1}). All the films were thermally annealed at 160 °C prior to the measurements.

Static water contact angles

Static water contact angles of the films were measured. The larger contact angles for the surface modified films compared with the PCBM film indicate that the surface energies of the films were lowered by surface segregation of bSi, nSi, and pSi.

Table S1. Static water contact angle on PCBM, bSi/PCBM, nSi/PCBM, and pSi/PCBM films. Values in parentheses are standard deviations. The films with SSM were prepared by spin-coating the mixed solution of PCBM (10 g L^{-1}) and the surface modifiers. The concentration of bSi, nSi and pSi are 0.88 g L^{-1}, 1.32 g L^{-1} and 1.36 g L^{-1}, respectively. All the films were thermally annealed at 160 °C.

**PCBM** (°)	**bSi/PCBM** (°)	**nSi/PCBM** (°)	**pSi/PCBM** (°)
78.9 (1.1)	94.1 (0.7)	97.2 (0.3)	103.3 (0.8)

XPS depth profiles

XPS depth profiles of the films with bSi, nSi, and pSi showed that the Si 2p peak of all the films decreased greatly after 6 s surface etching with an Ar^+ ion beam. This suggests that the oligosiloxane chains of bSi, nSi, and pSi segregated to the surface and a small amount remained in the bulk of the films.

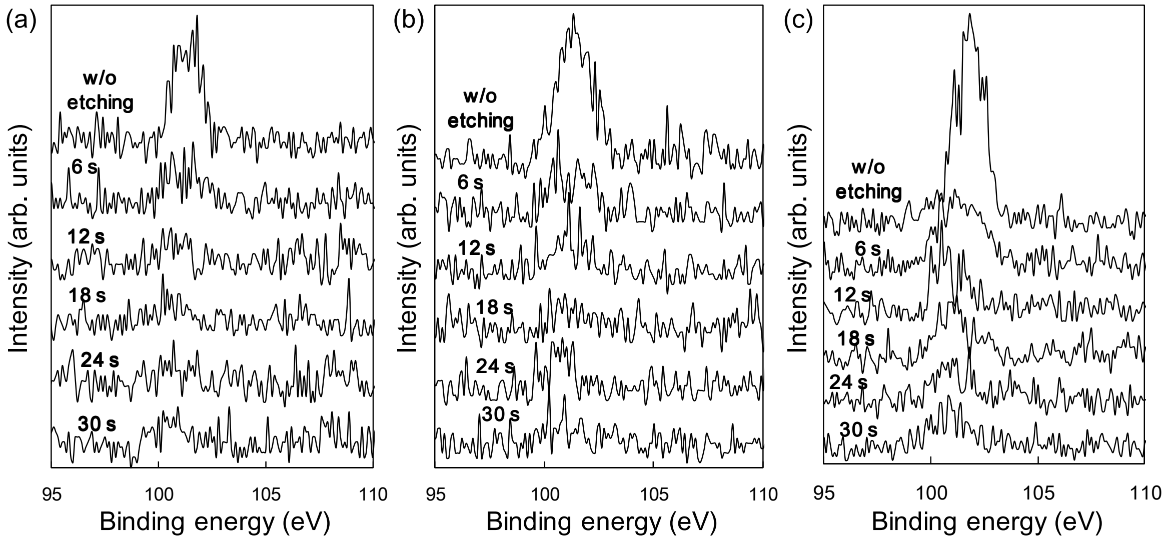

Figure S4. XPS depth profiles of (a) bSi/PCBM, (b) nSi/PCBM and (c) pSi/PCBM films. The films with SSM were prepared by spin-coating the mixed solution of PCBM (10 g L^{-1}) and the surface modifiers. The concentration of bSi, nSi and pSi are 0.88 g L^{-1}, 1.32 g L^{-1} and 1.36 g L^{-1}, respectively. All the films were thermally annealed at 160 °C. Surface etching with an Ar^+ ion beam (etching rate: 0.25 nm/s).

Angle-resolved XPS

The thickness of the oligosiloxane layer on the surface of the films was estimated by angle-resolved XPS (ARXPS) by using a uniform bilayer model that consists of oligosiloxane and fullerene layers. According to previous reports,[1]

$$\ln\left(\frac{I_{Si}X_C}{I_C X_{Si}}+1\right)=\frac{d}{\lambda\cos\theta} \qquad (1)$$

where I_{Si} and I_C are the intensities of Si 2p and C 1s peaks, λ is the attenuation length of photoelectrons, θ is the take-off angle of the measurements, and X_{Si} and X_{C} are the local concentrations of silicon and carbon atoms, respectively. We assume that the attenuation lengths of photoelectrons from C 1s and Si 2p are 3.0 nm because the kinetic energies of a photoelectron from C 1s (1205 eV) and Si 2p (1385 eV) are similar,[1] and that carbon atoms are present only in the fullerene layer in the model because the carbon atom density in the fullerene layer is much larger than in the oligosiloxane layer. X_{Si} was calculated from the number of silicon atoms and the length of oligosiloxane chain, which was obtained from optimized structure in the DFT calculation, as 3/0.45 in the bSi/PCBM film, 3/0.67 in the nSi/PCBM film, and 11/1.39 in the pSi/PCBM film. X_{C} was calculated from the number of carbons in PCBM/length of PCBM as 72/1.26.[2] Using eq. 1, a plot of ln ($I_{\mathrm{Si}}X_{\mathrm{C}}/I_C X_{\mathrm{Si}}$ + 1) as a function of 1/cos θ was fitted well by a straight line through the origin (Figure S5). These results indicate that the continuous surface segregated monolayers of bSi, nSi, and pSi formed on the surface of PCBM films. The thickness of the oligosiloxane layer (*d*) can be calculated from the slope of these lines. The results were 0.39 nm for the bSi/PCBM film, 0.69 nm for the nSi/PCBM film, and 0.86 nm for the pSi/PCBM film, whereas the calculated lengths of the oligosiloxane chains were 0.45, 0.67, and 1.39 nm, respectively. The larger difference in pSi might be attributed to the molecular weight distribution of the oligosiloxane and the different conformation of the flexible chains between the film and the optimized structure.

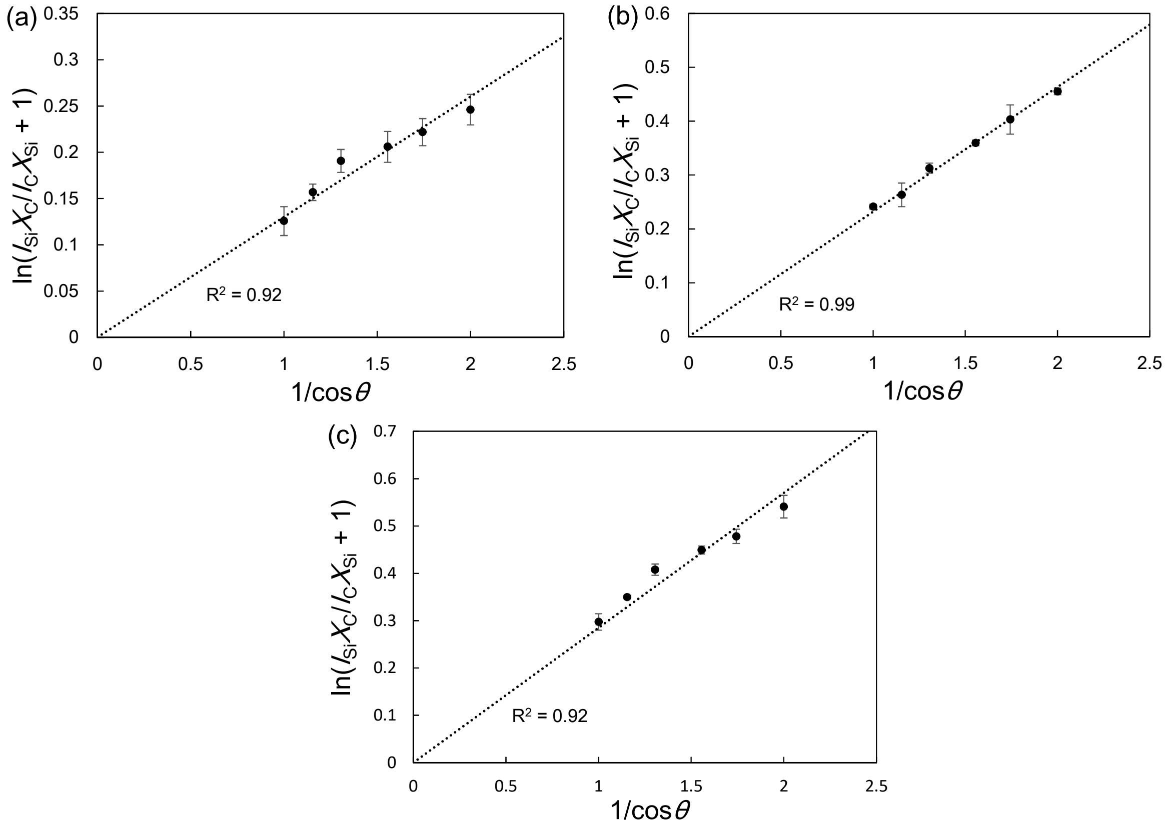

Figure S5. Plot of 1/cos θ vs ln ($I_{Si}X_C$/ I_CX_{Si} + 1) from Si and C peaks from the ARXPS spectra in the (a) bSi/PCBM, (b) nSi/PCBM, and (c) pSi/PCBM films. The films with SSM were prepared by spin-coating the mixed solution of PCBM (10 g L^{-1}) and the surface modifiers. The concentration of bSi, nSi and pSi are 0.88 g L^{-1}, 1.32 g L^{-1} and 1.36 g L^{-1}, respectively. The films were thermally annealed at 160 °C prior to the measurements. The lines indicate the best fit with eq. 1.

Elemental mapping

Elemental mapping of the PCBM films with bSi, nSi, and pSi was performed by scanning transmission electron microscopy (STEM) as shown in Figure S6. The results indicated that carbon and silicon atoms homogeneously dispersed at the surface of the films.

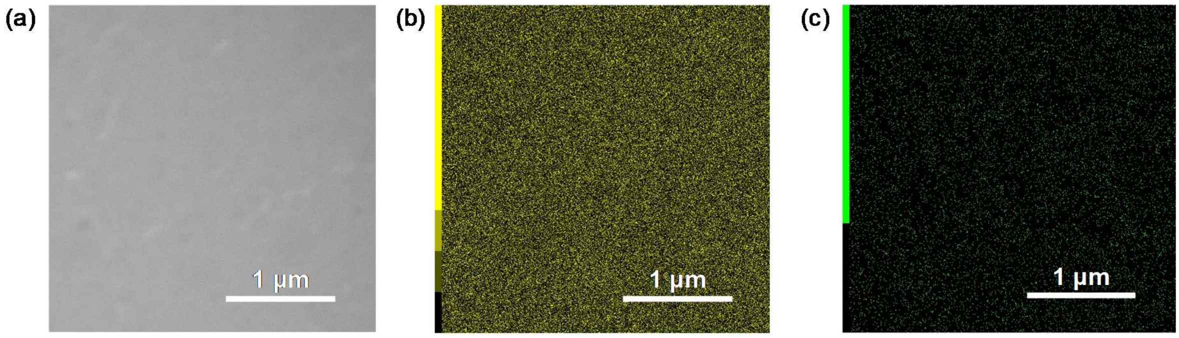

Figure S6. (a) STEM image of pSi/PCBM film. 2D mapping by energy dispersive X-ray spectrometry of (b) carbon and (c) silicon in the same area as in (a). The pSi/PCBM film were prepared by spin-coating the mixed solution of pSi (1.36 g L^{-1}) and PCBM (10 g L^{-1}). The film was thermally annealed at 130 °C.

Incident angle dependence of GIWAXS patterns

The critical angle of Si substrate (density: 2.33 g/cm^3) is 0.144°, whereas the critical angle calculated by the density of the PCBM film (1.49 g/cm^3) is 0.115°. GIWAXS patterns measured at an incident angle of 0.12° reflects the diffraction from the crystal in the bulk of the thin films. Figure S7 shows the incident angle dependence of GIWAX patterns for the annealed pSi/PCBM film. The patterns with the smaller incident angles showed smaller peak intensities without any change of the pattern, indicating that the diffraction patterns came from the bulk of the film and not only from the surface.

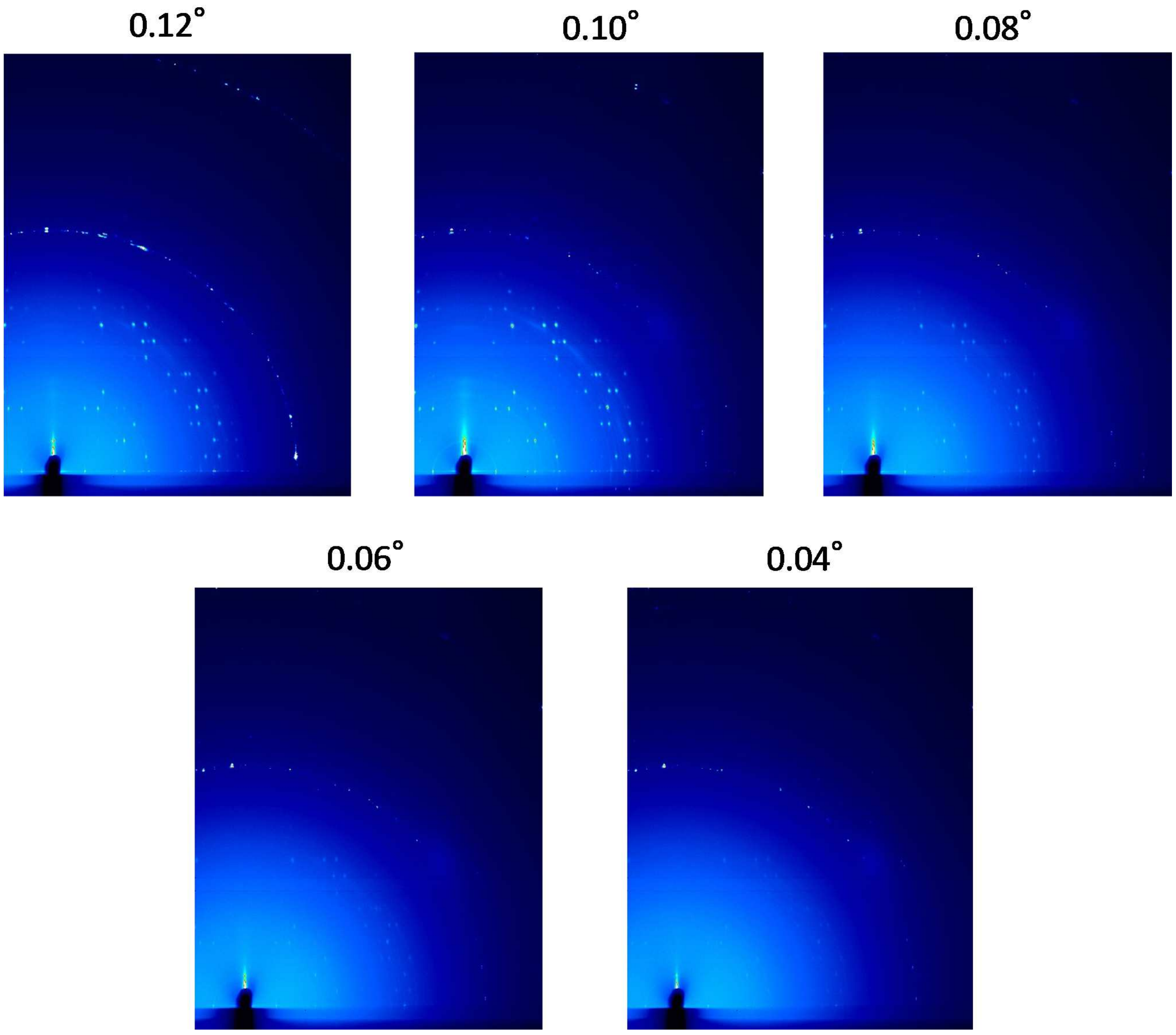

Figure S7. Incident angle dependence of GIWAXS patterns for the annealed pSi/PCBM film.

GIWAXS patterns

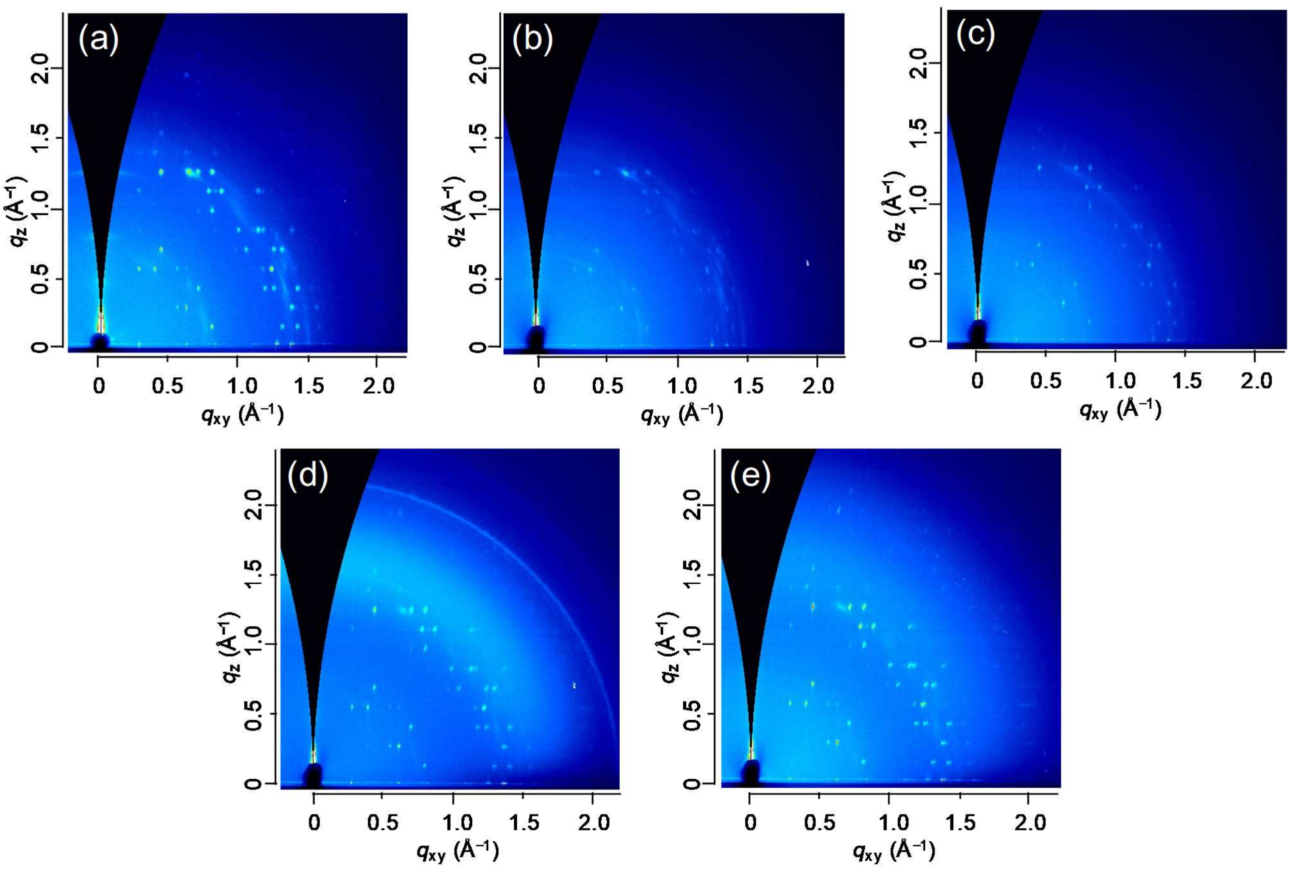

Figure S8. GIWAXS patterns of the (a) bSi/PCBM, (b) nSi/PCBM, (c) FC_8/PCBM on silicon wafers, (d) bSi/PCBM film on an ITO substrate, and (e) bSi/PCBM on a glass substrate on a silicon wafer. The films with SSM were prepared by spin-coating the mixed solution of PCBM (10 g L^{-1}) and the surface modifiers. The concentration of bSi, nSi, and FC_8 are 0.88 g L^{-1}, 1.32 g L^{-1} and 1.00 g L^{-1}, respectively. The films were thermally annealed at 160 °C.

TEM image of pSi/PCBM film

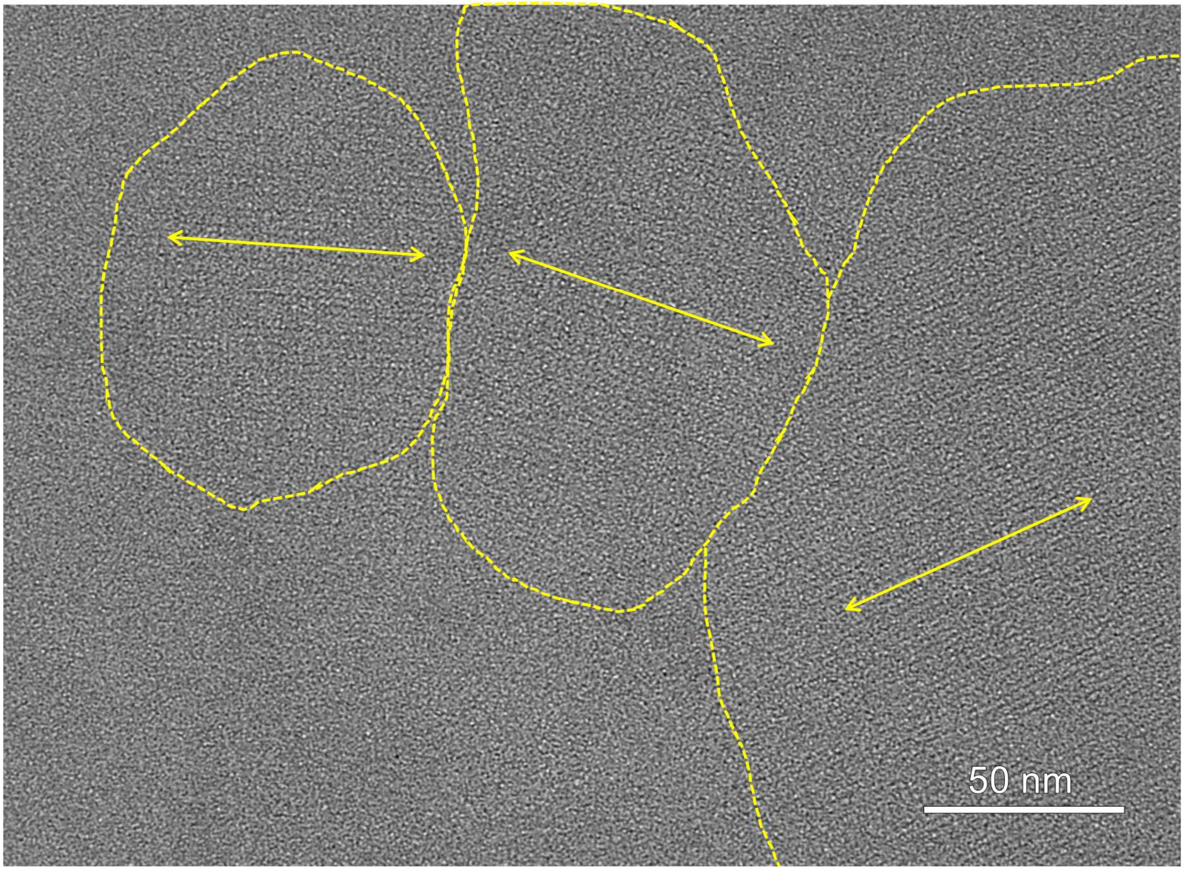

Figure S9. TEM image of pSi/PCBM film. The pSi/PCBM film were prepared by spin-coating the mixed solution of pSi (1.36 g L^{-1}) and PCBM (10 g L^{-1}). The pSi/PCBM film sample was transferred onto a Cu grid with a carbon supporting film by using CYTOP as a support layer. The pSi/PCBM film was thermally annealed at 130 °C before the film transfer. The arrows in the image indicate the directions of the crystal lattice and the broken lines indicate the areas of the crystals.

Size of the crystalline domains estimated by Scherrer equation

Mean size of the crystalline domains (τ) can be calculated by the following Scherrer equation:

$$\tau = \frac{K\lambda}{\beta cos\theta}$$

Where K is the shape factor (typical value: 0.9), λ is the X-ray wavelength (0.1 nm), β is the spectrum

broadening at half the maximum intensity (FWHM) of diffraction peaks, and θ is the Bragg angle. The plot profile of the GIWAXS patterns of the annealed pSi/PCBM film are shown in Figure S10. FWHM of 110 peak at 0.0217 rad is 0.000487 rad, therefore τ of 110 direction is calculated to be 185 nm. This value should be regarded as the lower limit for the crystal size because the grazing incident angle makes a large longitudinal footprint of the X-ray and causes the significant broadening of the peaks. Nevertheless, the value is in the same order with the observed domain sizes in the TEM image. (Figure S9).

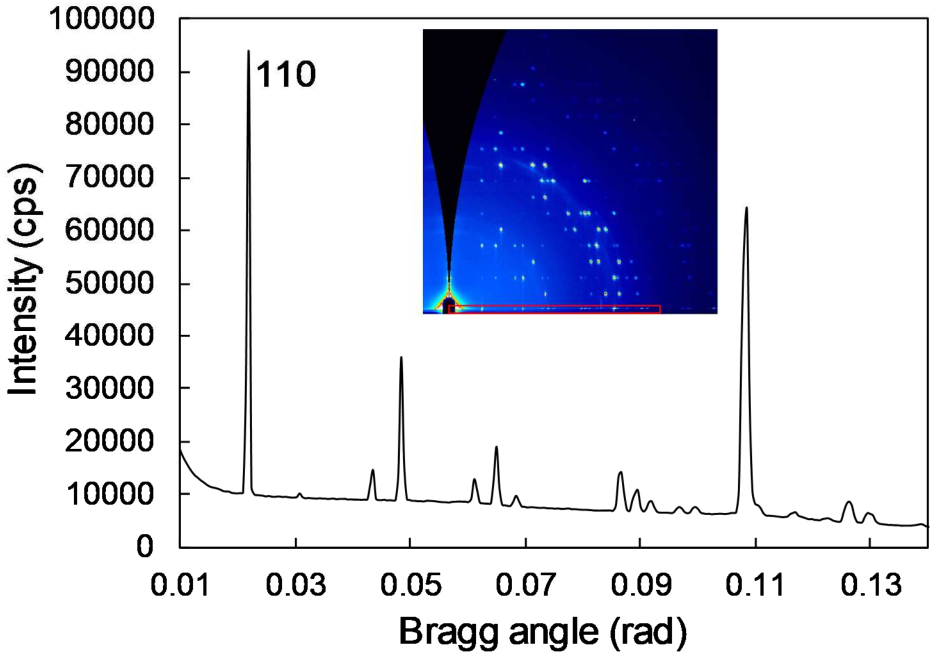

Figure S10. Plot profile of the GIWAXS patterns of the annealed pSi/PCBM film. The inset is the GIWAXS patterns of the annealed pSi/PCBM film (Figure 3b). The plot profile is calculated by integrating the area of the red rectangle on the GIWAXS patterns in the direction of *xy* axis.

Line profiles of AFM images

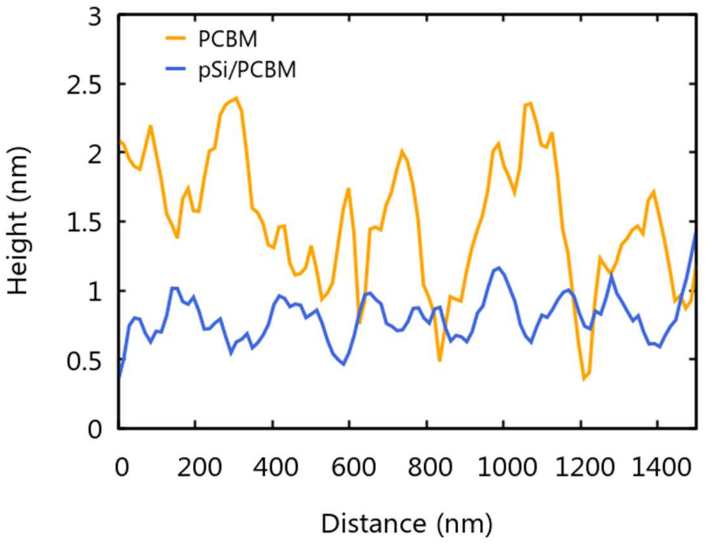

Figure S11. Line profiles of AFM images along the white lines in Figure 3g and h.

Crystal structure analysis of PCBM in SSM-induced phase

To determine the crystal system, the cell constant and the space group for the crystal structure, diffraction pattern calculator (DPC) toolkit was used on the GIWAXS image.[3] The unit cell was determined as tetragonal with $a = b = 32.73$, $c = 45.55$ Å and $V = 48795.6$ Å^3, which are consistent with the out-of-plane XRD and SAED patterns. Visual matching of the systematic extinction rules for the GIWAXS pattern narrows down the possible space group to $I4cm$, $I\bar{4}c2$ and $I4/mcm$. However, $I4/mcm$ has 32 general positions, therefore does not satisfy the requirement of $Z \sim 48$ and the density of 1.5-1.65 g cm^{-3} with any integer numbers of the independent molecules in the unit cell. $I4cm$ and $I\bar{4}c2$ have 16 general positions and satisfy $Z = 48$ with three crystallographically independent PCBM molecules in the unit cell. Therefore, we investigate these two space groups for the following analysis.

After the correction of the distortion due to the use of the planar image detector, the GIWAXS patterns

were converted to a series of lateral 1D profiles by using the plot profile function of ImageJ (NIH). The peaks in the 1D profiles are fitted by pseudo-Voigt functions by using PDXL software (Rigaku) and the peak intensities are extracted by the integrations of the peak area. In-plane data (*hk*0) was omitted from the analysis to avoid the effects of the Yoneda wings.[4] The intensity data were corrected with Lorentz and polarization factors considering the measurement geometry. Diffraction data of out-of-plane geometry, which cannot be measured in GIWAXS geometry, were separately collected with a $2\theta/\omega$ scan technique performed on an X-ray diffractometer (SmartLab, Rigaku) by using a parallel X-ray beam (Cu Kα1 radiation at 45 kV and 200 mA). The peaks were integrated, and the appropriate Lorentz and polarization corrections were applied to obtain the 00*l*-*I* dataset.

The structure was first solved by direct-space method with a simulated annealing algorithm implemented in SIR2014 software[5] using the *hkl*-I dataset from GIWAXS to explore the positions of the three independent PCBM molecules in the unit cell. Owing to the low resolution of the diffraction data, intermolecular anti-bumping constraints were imposed in the simulated annealing exploration runs. The runs are repeated until a solution with the lowest figure of merit (*R*-value) was obtained. Since the assumption of *I*4*cm* space group resulted in unrealistic porous structures with relatively high *R*-values, $I\bar{4}c2$ space group was adopted. The optimized structure was further refined by using SHELXLL-2016/6 software.[6] The GIWAXS and the out-of-plane data were scaled by batch scale-factor refinements. The C_{60}, phenyl and ester moieties were treated as rigid bodies, and the bond distances and angles of the other moieties were strongly constrained with those of the reported single-crystal PCBM structure. GIWAXS pattern was simulated by using a program made by T. Koganezawa

from the structure with the lowest figure of merit (R = 0.3858 for 136 reflections with $I > 2\sigma (I)$, attached CIF). The solution can reproduce the observed pattern well (Figure 4a and b).

The mosaicity of the crystal in the out-of-plane direction has been estimated to be 0.95(2)° from the rocking-curve measurement on the 0 0 10 reflection. We have also analyzed the Bragg spots of 419, 545 and 723, which were isolated from other spots and had strong intensities on the 2D GIWAXS image. On the 2D image, the omega direction (i.e. scanning axis of rocking curve measurement) corresponds to a tangent of the circle centered on the direct beam at each Bragg spot. 1D-profiles of the three spots were extracted along the omega-directions, and the profiles were fitted by a pseudo-Voight function. The mosaicity of the sample was estimated to be 1.49(8)°, 1.62(5)° and 1.62(5)° for the 419, 545 and 723 reflections, respectively, from the integration widths along the omega direction.

XRD patterns after annealing at different temperatures

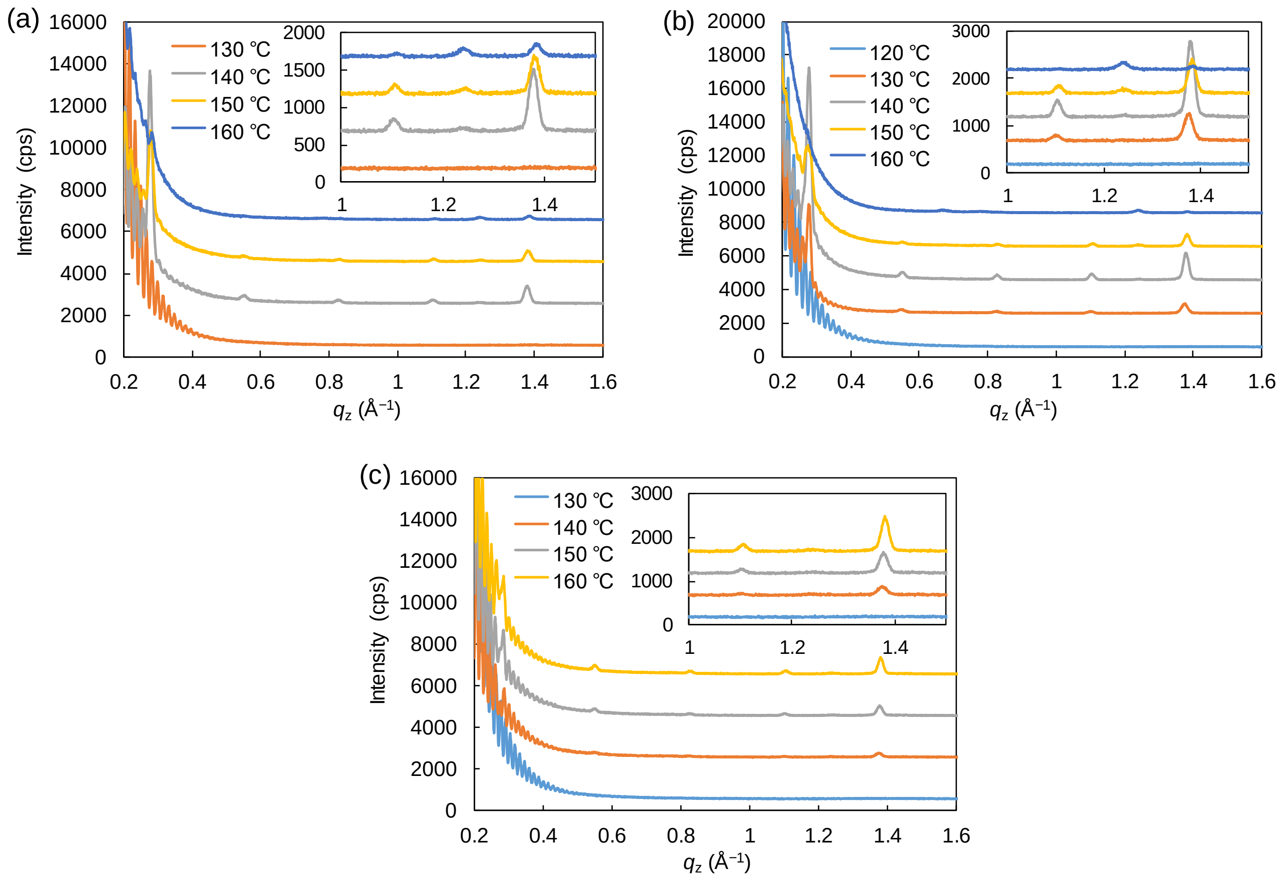

Figure S12. Out-of-plane XRD pattens of (a) bSi/PCBM, (b) nSi/PCBM and (c) FC_8/PCBM films on silicon wafers, which were thermally annealed at different temperatures. The films with SSM were prepared by spin-coating the mixed solution of PCBM (10 g L^{-1}) and the surface modifiers. The concentration of bSi, nSi and FC_8 are 0.88 g L^{-1}, 1.32 g L^{-1} and 1.00 g L^{-1}, respectively. The insets show magnified views. The patterns are shifted in the *y*-axis for clarity.

F:C atomic ratio at the surface of FC_8/PCBM films

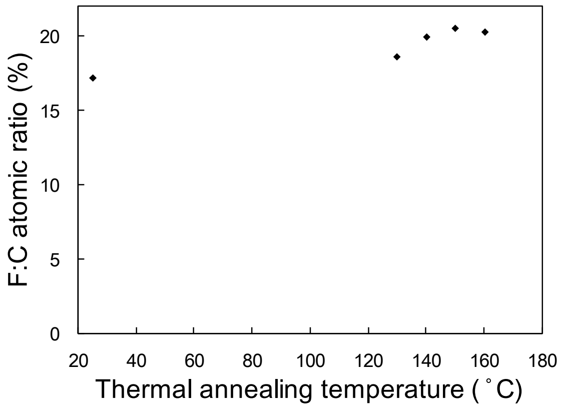

Figure S13. F:C atomic ratio on the surfaces of the FC_8/PCBM films measured by XPS plotted as a function of the thermal annealing temperature. The FC_8/PCBM films were prepared by spin-coating the mixed solution of PCBM (10 g L^{-1}) and FC_8. The concentration of FC_8 is 1.00 g L^{-1}, respectively.

Nano *I-V* curve and space charge-limited current model analysis in c-AFM

I-V curves were measured at 10 different points by c-AFM. Figure S14 shows all the *I-V* curves and measurement points. The electron mobility was calculated by using the modified SCLC model for c-AFM in the case of electron injection from the substrate.[7] The modified SCLC model is expressed by

$$J = \frac{3\varepsilon_0\varepsilon_r\mu V^2}{2L^3} \qquad (2)$$

where J is the current density, ε_0 is the vacuum dielectric constant, ε_r is the relative dielectric constant (PCBM: 3.9),[8] μ is the electron mobility, V is applied voltage, and L is the film thickness (40 nm). Current density was calculated from the measured current and tip-sample contact area. The radius of the tip-sample contact (A) is given by Hertzian mechanics as[9]

$$A = \frac{RF}{3K} \qquad (3)$$

where R is the radius of the tip (25 nm), F is the contact force of the tip on the sample surface (2

nN), and K is the elastic modulus of PCBM (3.0 GPa).[10]

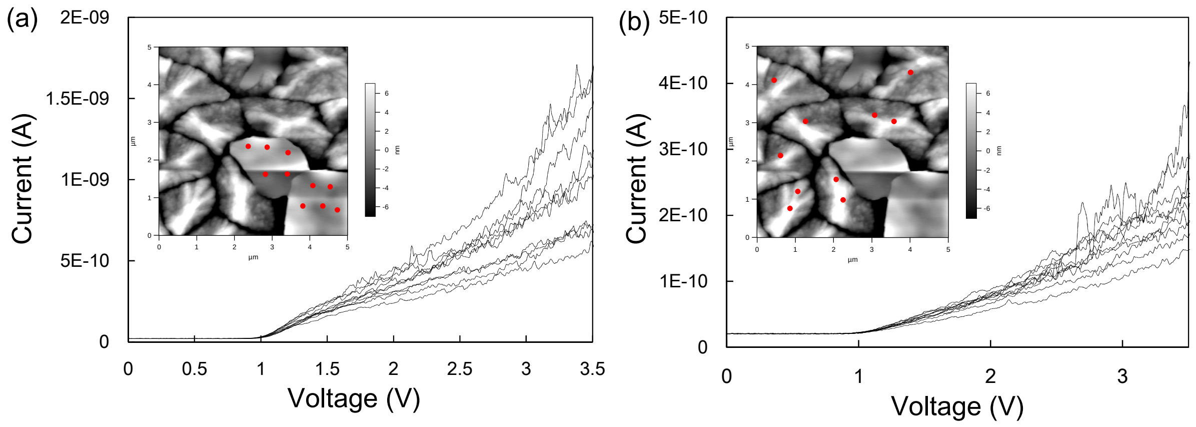

Figure S14. Nano *I-V* curves at 10 different points on (a) surface-segregated monolayer (SSM)-induced crystal and (b) other positions. Measured points are shown as red dots in the topography images (inset). The films were thermally annealed at 160 °C.

Table S2. Parameters in the fitting curves ($I = 10^b V^a$).

	SSM crystal	Normal crystal
Slope *a*	2.04	2.03
Intercept *b*	−10.08	−10.72

References

1 Ton-That, C., Shard, A. G. & Bradley, R. H. Thickness of spin-cast polymer thin films determined by angle-resolved XPS and AFM tip-scratch methods. *Langmuir* **16**, 2281-2284, doi:10.1021/la990605c (2000).

2 Izawa, S., Nakano, K., Suzuki, K., Hashimoto, K. & Tajima, K. Dominant Effects of First Monolayer Energetics at Donor/Acceptor Interfaces on Organic Photovoltaics. *Advanced Materials* **27**, 3025-3031, doi:10.1002/adma.201500840 (2015).

3 Hailey, A. K., Hiszpanski, A. M., Smilgies, D. M. & Loo, Y. L. The Diffraction Pattern Calculator (DPC) toolkit: a user-friendly approach to unit-cell lattice parameter identification of two-dimensional grazing-incidence wide-angle X-ray scattering data. *J. Appl. Crystallogr.* **47**, 2090-2099, doi:10.1107/S1600576714022006 (2014).

4 Yoneda, Y. Anomalous Surface Reflection of X Rays. *Physical Review* **131**, 2010-2013, doi:10.1103/PhysRev.131.2010 (1963).

5 Burla, M. C. *et al.* Crystal structure determination and refinementviaSIR2014. *J. Appl. Crystallogr.* **48**, 306-309, doi:10.1107/s1600576715001132 (2015).

6 Sheldrick, G. M. Crystal structure refinement with SHELXL. *Acta Crystallogr C Struct Chem* **71**, 3-8, doi:10.1107/S2053229614024218 (2015).

7 Woellner, C. F., Freire, J. A., Guide, M. & Nguyen, T. Q. The theoretical current-voltage dependence of a non-degenerate disordered organic material obtained with conductive atomic force microscopy. *Journal of Chemical Physics* **135**, doi:10.1063/1.3626871 (2011).

8 Jahani, F., Torabi, S., Chiechi, R. C., Koster, L. J. A. & Hummelen, J. C. Fullerene derivatives with increased dielectric constants. *Chemical Communications* **50**, 10645-10647, doi:10.1039/c4cc04366a (2014).

9 Wold, D. J. & Frisbie, C. D. Fabrication and characterization of metal-molecule-metal junctions by conducting probe atomic force microscopy. *Journal of the American Chemical Society* **123**, 5549-5556, doi:10.1021/ja0101532 (2001).

10 Awartani, O. *et al.* Correlating Stiffness, Ductility, and Morphology of Polymer:Fullerene Films for Solar Cell Applications. *Advanced Energy Materials* **3**, 399-406, doi:10.1002/aenm.201200595 (2013).
